# Lysophosphatidylcholine homeostasis via the Lands cycle regulates root growth in Arabidopsis

**DOI:** 10.1093/pcp/pcaf174

**Published:** 2026-01-12

**Authors:** Liping Wang, Michael Kazachkov, Li Qin, Yuxing Niu, Yangdou Wei, Qiang Li, Jitao Zou

**Affiliations:** Department of Molecular and Cellular Biology, University of Guelph, 50 Stone Road East, Guelph, Ontario, N1G 2W1, Canada; National Research Council-Saskatoon Aquatic and Crop Resource Development Research Centre, National Research Council of Canada, 110 Gymnasium Place, Saskatoon, SK, Canada; Department of Biology, University of Saskatchewan, 105 Administration Place, Saskatoon, SK, Canada; Department of Plant Science, 202 Tyson Building, The Pennsylvania State University, University Park, PA, USA; Department of Biology, University of Saskatchewan, 105 Administration Place, Saskatoon, SK, Canada; National Key Laboratory of Crop Genetic Improvement, Huazhong Agricultural University, 1 Shizishan Street, Hongshan District, Wuhan, China; National Research Council-Saskatoon Aquatic and Crop Resource Development Research Centre, National Research Council of Canada, 110 Gymnasium Place, Saskatoon, SK, Canada; Department of Plant Science, 202 Tyson Building, The Pennsylvania State University, University Park, PA, USA

**Keywords:** Arabidopsis, ER membrane, Lands cycle, lysophosphatidylcholine acyltransferase, lipid homeostasis, root growth

## Abstract

Lysophosphatidylcholine acyltransferase (LPCAT) is a key enzyme in the Lands cycle that mediates the interconversion of phosphatidylcholine (PC) and lysophosphatidylcholine (LPC). A proper balance between LPC and PC is essential for phospholipid turnover and membrane homeostasis. In this study, we show that the Arabidopsis *lpcat* double mutant exhibits an altered LPC/PC ratio in root tissues, reflecting a compromised Lands cycle. Using a chemical phenocopying strategy, we found that root growth in the *lpcat* mutant was more sensitive than that the wild type to lyso-platelet-activating factor (lysoPAF), which is a structural analog of LPC. This growth defect was largely rescued when the lysoPAF treatment was combined with ONO-RS-082, which is a phospholipase A_2_ inhibitor. Subcellular localization using green fluorescent protein (GFP) fusions demonstrated that both LPCAT1 and LPCAT2 localize to the endoplasmic reticulum (ER) membrane. Furthermore, an RNA-seq analysis of root tissues from the *lpcat* mutant revealed transcriptional changes indicative of an unfolded protein response in the ER and altered endomembrane trafficking. Together, our results demonstrate that ER-localized LPCATs play a pivotal role in maintaining root growth when plants encounter conditions that require adjustments in lipid homeostasis.

## Introduction

Glycerolipids form the structural foundation of cellular membranes, and their composition critically determines the functional properties of membrane systems. The most predominant structural element in the eukaryotic cell membrane is phosphatidylcholine (PC). In plants, PC is primarily synthesized in the endoplasmic reticulum (ER) and is subsequently distributed to other membrane systems, placing it at a vital intersection in glycerolipid metabolism. PC serves multiple essential roles: it is the primary substrate for ER-localized fatty acid desaturation (Li-Beisson et al. 2013, Shockey et al. 2016); it provides a primary platform for acyl exchange, and it acts as an intermediate in the biosynthesis of diacylglycerol and triacylglycerol (Bates et al., 2013; Hu et al., 2012).

The PCs in membrane systems also undergo acyl remodeling by a process known as the Lands cycle (Hill and Lands 1970). PC remodeling involves the interconversion between PC and lysophosphatidylcholine (LPC) through two enzymatic steps: phospholipase A_2_ (PLA_2_) deacylates PC to produce LPC and a free fatty acid, and LPC acyltransferase (LPCAT) reacylates LPC with a fatty acyl-CoA to regenerate PC (Wang et al. 2012, 2014, Bates et al. 2013, Lager et al. 2013). Importantly, the fatty acid released from PC by PLA_2_ can differ from the acyl species incorporated by LPCAT, thereby altering the fatty acid composition of PC without affecting its overall steady-state level. For this reason, the PC remodeling process is also referred to as acyl editing.

The enzyme LPCAT plays a pivotal role in PC remodeling by enabling continuous acyl exchange between PC and the acyl-CoA pool (Stymne and Stobart 1984, Bates et al. 2007, 2013). In leaf tissues, LPCAT also participates in incorporating newly synthesized fatty acids exported from chloroplasts into PC, thereby supporting the trafficking of nascent fatty acids between plastidial and extraplastidial lipid pools (Bates et al. 2009, Xu and Shanklin 2016). Through these activities, LPCAT maintains dynamic acyl exchange between organelles and supports overall membrane lipid homeostasis (Wang et al. 2012, Jasieniecka-Gazarkiewicz et al. 2016).

Although most studies of PC remodeling have focused on acyl editing, a less appreciated but equally critical aspect is its role in regulating the LPC/PC ratio. In Arabidopsis, two LPCAT isoforms, namely LPCAT1 and LPCAT2, are expressed throughout development. Previous studies have shown that the disruption of acyl remodeling in the *lpcat* double mutant, while not causing major changes in glycerolipid fatty acid profiles, leads to LPC overaccumulation in both leaves and seeds (Wang et al. 2012, 2014). LPCAT deficiency also triggers accelerated PC turnover. The *lpcat* mutant exhibited elevated transcript levels of several lipases and increased accumulation of extracellular free choline in the water-soluble fraction (Wang et al. 2012). Surprisingly, the cellular PC content was not reduced. The steady-state level of PC was maintained through a compensatory increase in PC synthesis. This was confirmed by a choline feeding experiment, which showed that *de novo* PC synthesis in the mutant occurred at a rate 1.5- to 1.8-fold higher than in wild-type plants (Wang et al. 2012).

The distinctive inverted-cone shape of LPC contrasts with the cylindrical geometry of PC. These structural differences lead to distinct effects on membrane organization, curvature generation, and functional dynamics. LPC promotes positive membrane curvature, whereas PC favors the formation of lamellar bilayers and stabilizes the planar membrane structure (Vamparys et al. 2013, McMahon and Boucrot 2015). Critical steps in membrane dynamics, such as budding, vesicle fission, and fusion, require the generation of curvature and the relaxation of surface tension, which are processes that often occur in tandem. Most studies in this area have focused on protein components that drive membrane deformation and vesicle trafficking, yielding detailed insight into how coat proteins sculpt membrane architecture. Growing evidence, however, indicates that lipids also make an active and essential contribution to these processes. In particular, excess LPC has been shown to induce mechanical deformations and packing defects in model membranes (Jackson 2018) and to interfere with membrane protein stability.

Here, we investigated the physiological and molecular consequences of LPCAT deficiency in *Arabidopsis roots*. We present direct evidence that the Arabidopsis LPCATs are ER-resident proteins. Using a combination of genetic, chemical, and transcriptomic approaches, we show that a loss of LPCAT function increases the LPC/PC ratio, triggers ER stress-associated gene expression, and compromises root growth under lipid-perturbing conditions. Together, our findings reveal that ER-localized LPCATs play a role in root growth, highlighting lipid remodeling and LPC/PC homeostasis as underexplored regulators of plant growth and development.

## Results

### *Lpcat* mutant roots exhibit elevated LPC levels and altered acyl composition

**Figure 1:**
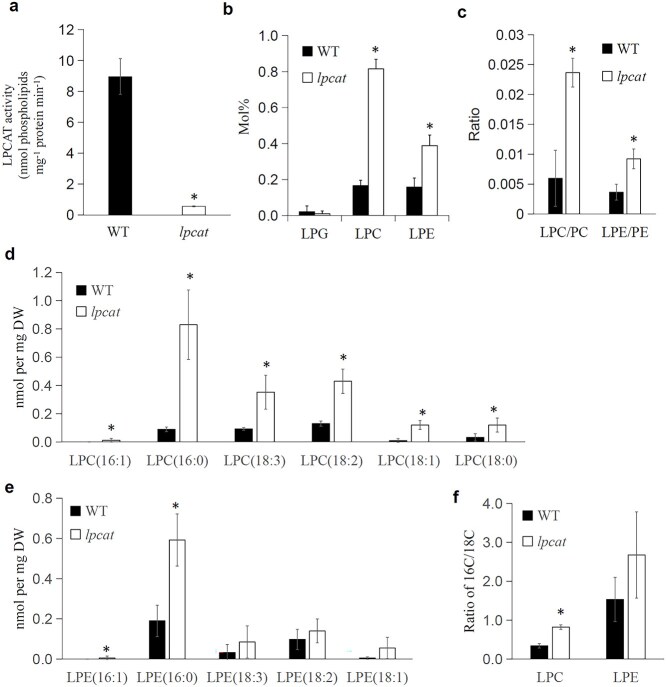
LPCAT deficiency alters lysophospholipid metabolism in roots. (a) LPCAT enzyme activity in microsomal fractions from roots. Data represent the means ± SD of three independent biological replicates. (b) Total lysophospholipid content expressed as nmol of lipid per mg of dry weight (DW). (c) Ratios of LPC/PC and LPE/PE in WT and *lpcat*-mutant roots. (d, e) Quantification of individual lysophospholipid species (nmol of lipid per mg of DW). (f) Ratios of 16C to 18C lysophospholipids in WT and *lpcat*-mutant roots. In (b–f), values represent the means ± SD of five independent biological replicates. Asterisks indicate statistically significant differences relative to the WT (Student’s *t*-test, *P* < 0.05).

We assessed LPCAT activity in the root tissues of the *lpcat* double mutant propagated on Murashige Skoog (MS) medium. Consistent with results from above-ground tissues (Wang et al. 2012, 2014), the LPCAT activity in the *lpcat-*mutant roots was decreased to approximately 1/20 of that of wild type (WT) levels (Fig. 1a). A comparison of WT tissues revealed that the LPCAT activity in roots was almost 10 times higher than in developing seeds (Wang et al. 2012), highlighting its functional importance in roots.

Lipidomic analyses showed that most glycerolipid species and their fatty acid compositions remained largely unchanged in the *lpcat* roots (Supplementary Fig. S1; Supplementary Data Set S1). However, the LPC and lysophosphatidylethanolamine (LPE) levels were elevated by three- and two-fold, respectively, relative to the WT (Fig. 1b). Correspondingly, the LPC/PC and LPE/PE ratios increased to four- and 2.5-fold higher than those of the WT (Fig. 1c), underscoring the importance of the Lands cycle in maintaining these lipid ratios.

We further examined the molecular species of LPC and LPE, focusing on the 16C versus 18C species. Almost all LPC species and 16:0-LPE were substantially increased in the mutant roots (Fig. 1d and e). In WT roots, the 16C/18C LPC ratio was 0.33, whereas in the *lpcat* roots, it increased to 0.81 (Fig. 1f). Given that 16C fatty acids in ER-derived glycerolipids are primarily located at the *sn-1* position, this perturbed metabolite profile is consistent with the LPCAT’s role in primarily mediating the *sn-2* acylation of LPC (Lager et al. 2013).

### Root development in the *lpcat* mutant is hypersensitive to lysoPAF, a structural analog of LPC

**Figure 2:**
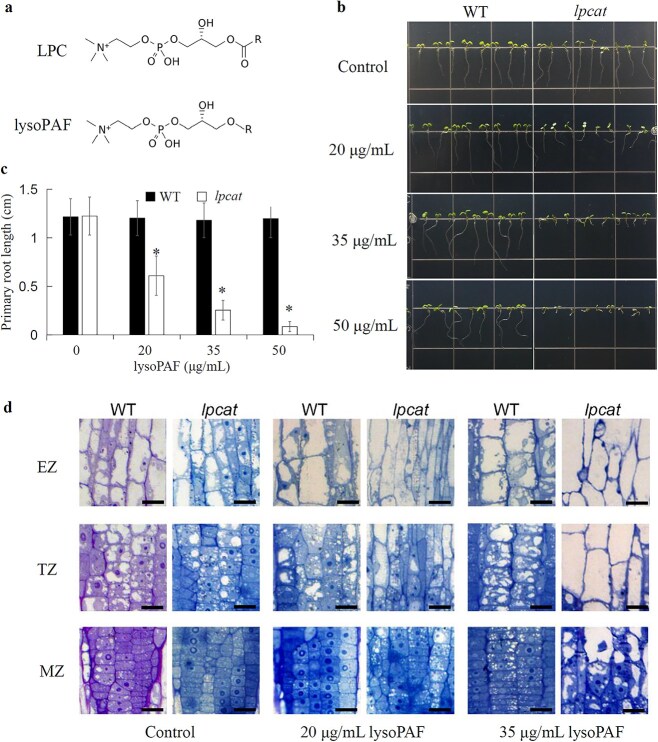
*Lpcat* roots are hypersensitive to lysoPAF. (a) Chemical structures of LPC and its alkyl analog, lysoPAF. (b) Seedlings grown on an MS medium supplemented with 0 *μ*g/mL (control), 20, 35, or 50 *μ*g/mL of lysoPAF under continuous light. Images were taken 4 days after germination. (c) Primary root length was quantified using ImageJ. Data are the means ± SD (n = 10–15). Asterisks denote significant differences compared with the WT (*P* < 0.05; two-tailed Student’s *t*-test). (d) Light microscopy of root cells in the MZ, TZ, and EZ. Scale bars = 10 *μ*m.

The Arabidopsis *lpcat* mutant, despite having an approximately four-fold higher cellular LPC/PC ratio than the WT (Fig. 1b), does not exhibit obvious developmental defects under standard growth conditions (Wang et al. 2012, 2014). When examined on MS media supplemented with exogenous LPC, the root growth of both WT and the *lpcat* seedlings was unaffected at 30 *μ*g/mL of LPC. At 90 *μ*g/mL, the roots appeared slightly wavy, but they showed no discernible differences between the genotypes. LPC at 300 *μ*g/mL severely inhibited root growth in both the WT and *lpcat* (Supplementary Fig. S2). The absence of a distinct growth phenotype in the *lpcat* mutant, and the observation that the WT also exhibited growth inhibition at high LPC levels, suggest that compensatory metabolic pathways likely buffered the effects of elevated LPC, thereby masking any *lpcat*-specific phenotype. Specifically, LPC can undergo further deacylation by lysophospholipases (LysoPL). Multiple isoforms of lysophospholipase (LysoPL) are present in *Arabidopsis*, which could detoxify LPC (Gao et al. 2010, Miao et al. 2019) by cleaving the carboxylic ester bond, releasing a fatty acid and glycerophosphocholine (GPC). We, thus, took a chemical biology approach by supplementing lyso-platelet-activating factor (lysoPAF), which is an alkyl homolog of LPC (Fig. 2a), in the growth media. LysoPAF shares the same geometrical shape as LPC; it can be efficiently acylated by LPCAT to generate a cylindrical phospholipid; but due to the ether linkage at the *sn-1* position, lysoPAF is recalcitrant to breakdown by lysoPL (Robinson and Snyder 1985). When grown on MS medium supplemented with lysoPAF, the *lpcat* seedlings exhibited a pronounced hypersensitivity to lysoPAF. The primary roots of both the WT and *lpcat* reached about 1 cm after 4 days under continuous light without lysoPAF. At 20 *μ*g/mL of lysoPAF, the *lpcat* roots were reduced to about two-thirds of the length of the WT; and at 35 *μ*g/mL, the *lpcat* root length was further reduced to only one-third of the WT. At 50 *μ*g/mL, root growth in the *lpcat* mutant was nearly arrested (Fig. 2b and c).

We next performed semi-thin transverse sectioning of the roots and examined them by light microscopy, focusing on developmental zones that were previously defined as: the meristem zone (MZ, up to 0.15 mm from the root tip), the transition zone (TZ, about 0.30 mm), and the elongation zone (EZ, 0.45−2 mm) (Birnbaum et al. 2003). In the absence of lysoPAF, the *lpcat* root cells were largely indistinguishable from the WT in all three zones (Fig. 2d, left two panels). At 20 *μ*g/mL of lysoPAF, the WT roots showed no apparent cellular changes, whereas the *lpcat* roots exhibited excessive vacuolation starting from the MZ (Fig. 2d, middle two panels). At 35 *μ*g/mL of lysoPAF, large vacuoles were prominent in the MZ of the *lpcat* mutant, and vacuolation in the TZ reached levels comparable with those in the EZ (Fig. 2d, right two panels).

### Inhibition of PLA_2_ alleviates lysoPAF-induced hypersensitivity in the *lpcat* root growth

**Figure 3:**
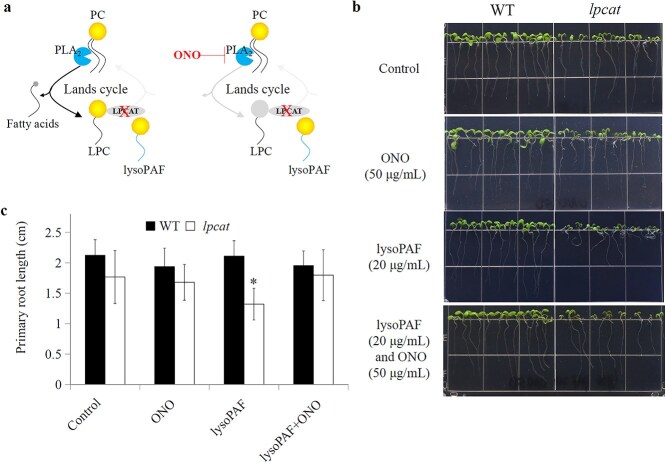
The phospholipase A_2_ inhibitor ONO alleviates the lysoPAF-induced inhibition of root growth. (a) A schematic representation of the ONO-mediated inhibition of the deacylation step in the Lands cycle. (b) The effects of the combined application of lysoPAF and ONO on root growth. (c) The primary root length was quantified using ImageJ. Data are presented as the means ± SD (n = 15). Asterisks denote significant differences compared with the WT (*P* < 0.05; Student’s *t*-test).

The Lands cycle operates with the sequential steps of PC deacylation by phospholipase A_2_ (PLA_2_) and LPC reacylation through LPCATs. In the *lpcat* mutant, the reacylation step is impaired, leading to an accumulation of LPC and an elevated LPC/PC ratio. We reasoned that if the lysoPAF-induced root growth phenotype arises directly from an imbalance in the Lands cycle, i.e. specifically from excessive LPC relative to PC, then inhibiting the deacylation step with PLA₂ should alleviate the phenotype. To test this, we applied a chemical, ONO-RS-082 (2-[*p*-amylcinnamoyl] amino-4-chlorobenzoic acid, C_21_H_22_ClNO_3_, hereafter referring to as ONO), which is a phospholipase A_2_ antagonist (de Figueiredo et al. 1998) that has been shown to inhibit at least three of the four *Arabidopsis* PLA_2_ isoforms in Arabidopsis (Lee et al. 2010) (Fig. 3a). ONO alone had no noticeable effect on growth or development under our experimental conditions (Fig. 3b and c). When combined with 20 *μ*g/mL of lysoPAF, ONO (50 *μ*g/mL) effectively suppressed the short root growth phenotype of the *lpcat* mutant (Fig. 3b and c). These results indicate that the hypersensitivity of the *lpcat* mutant to lysoPAF was likely a consequence of an impaired Lands cycle (Hill and Lands 1970), although secondary metabolic adjustments cannot be ruled out.

### Arabidopsis LPCATs are ER resident proteins

**Figure 4:**
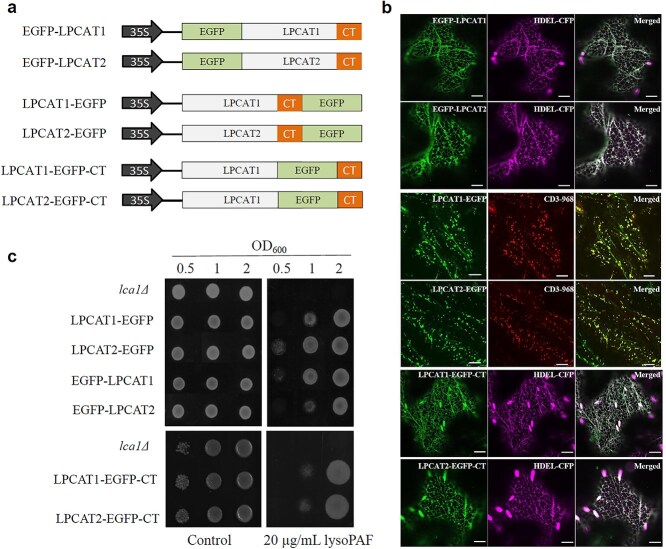
Cellular localization of *Arabidopsis* LPCAT proteins. (a) Schematic diagrams of vectors for EGFP fusion constructs with LPCAT1 or LPCAT2: EGFP-LPCAT, with EGFP fused to the N-terminus; LPCAT-EGFP, with EGFP fused after the C-terminal ER retention motif “RKEE”; and LPCAT-EGFP-CT, with EGFP inserted immediately before the “RKEE” motif. (b) Confocal microscopy images showing the subcellular localization of EGFP-LPCAT fusion proteins. Upper panel: EGFP-LPCAT co-localized with the luminal ER marker HDEL-CFP. Middle panel: LPCAT-EGFP co-localized with the Golgi marker CD3–968. Lower panel: LPCAT-EGFP-CT co-localized with HDEL-CFP in the ER. Scale bars = 20 *μ*m. (c) A functional assessment of LPCAT activity for all constructs in the yeast *lca1Δ* mutant, which lacks the LPCAT homolog. Yeast cells were grown in an induction medium and diluted to OD_600_ ₀₀ = 0.5, 1, and 2. Approximately 5 *μ*L of each dilution was spotted onto SC-Ura plates (control) or SC-Ura plates supplemented with 20 *μ*g/mL of lysoPAF. The assay for LPCAT-EGFP-CT was performed separately from the other constructs; a corresponding control is shown in the lower panel.

LPCATs belong to the membrane-bound O-acyltransferase (MBOAT) superfamily, whose members share a conserved structural fold characteristic of Membrane-bound O-acyltransferases (MBOATs) enzymes and are predominantly, though not exclusively, integral ER membrane proteins. The precise subcellular localization of the Arabidopsis LPCATs remains to be fully established. We generated enhanced green fluorescent protein (EGFP) fusion constructs with EGFP fused either to the N-terminus (EGFP-LPCAT) or the C-terminus (LPCAT-EGFP) (Fig. 4a). In transgenic plants expressing EGFP-LPCAT, fluorescence was primarily localized to the ER, as signals from the ER marker HDEL-CFP merged with those from the LPCATs (Fig. 4b, upper panel). By contrast, the LPCAT-EGFP plants exhibited fluorescence both in the ER and in small, punctuate structures, which co-localized with the Golgi marker CD3-968 (Fig. 4b, middle panel).

Both Arabidopsis LPCATs have a putative C-terminal ER retention motif, “RKEE.” To determine whether the C-terminal GFP fusion in the LPCAT-EGFP constructs masked the putative RKEE motif and affected ER retention, we generated versions in which EGFP was positioned upstream of this motif (LPCAT-EGFP-CT) (Fig. 4a). As expected, the fluorescence from these constructs was largely restricted to the ER, as confirmed by a complete overlap with the ER marker (Fig. 4b, lower panel), thereby validating the ER localization of the LPCATs. Across all constructs, LPCTA1 and LPCAT2 displayed similar intracellular localization patterns.

To verify that all LPCAT and EGFP fusion proteins were enzymatically active, we expressed the fusion constructs in the yeast *lpcat* (∆*lca1*) mutant, which is a strain known to be sensitive to lysoPAF (Chen et al. 2007, Zheng et al. 2012). All constructs, regardless of the EGFP position, complemented the yeast mutant, demonstrating that they retained functional LPCAT activity (Fig. 4c).

The root growth sensitivity of the *lpcat* mutant to lysoPAF provided an opportunity to examine if the ER-localized LPCAT GFP-fusions are functional in suppressing lysoPAF sensitivity. We introduced the enzymatically active LPCAT1-EGFP and LPCAT2-EGFP into the *lpcat* mutant. Positive transgenic lines were tested for root growth in the presence of lysoPAF. Transgenic lines expressing either LPCAT1 or LPCAT2 restored the root growth of the *lpcat* mutant under lysoPAF treatment (Supplementary Fig. S3). These results indicate that an ER-localized LPCAT is sufficient to rescue the *lpcat* root growth sensitivity to lysoPAF. They also confirmed that the lysoPAF sensitivity observed in the *lpcat* mutant was caused by the loss of LPCAT function itself, and not by other background mutations in the Salk lines.

### Transcriptome changes signify ER stress and impaired endomembrane trafficking in *lpcat*

**Figure 5:**
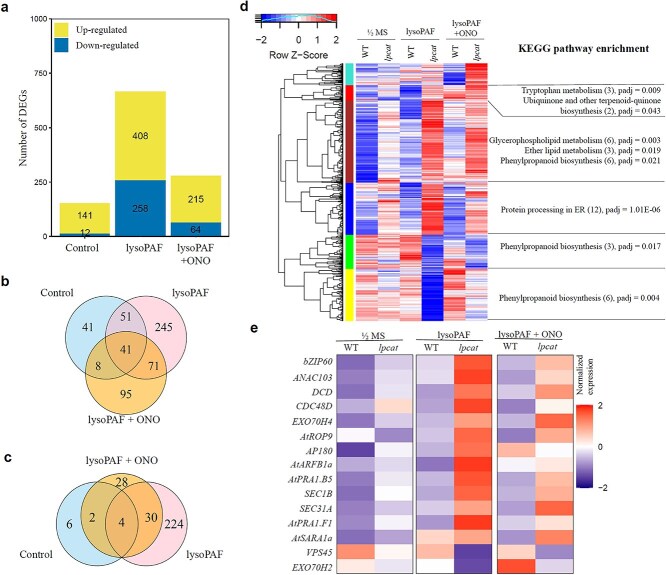
Transcriptomic alterations in the roots of the WT and the *lpcat* mutant under chemical treatments. WT and *lpcat* seedlings were grown on a ½ MS medium (control), ½ MS supplemented with 20 *μ*g/mL of lysoPAF (lysoPAF), or ½ MS supplemented with 20 *μ*g/mL of lysoPAF and 50 *μ*g/mL of ONO-RS-082 (lysoPAF + ONO). (a) The total number of DEGs between the WT and *lpcat*. (b, c) Venn diagrams showing the overlap of upregulated (b) and downregulated (c) DEGs. (d) Hierarchical clustering of DEGs between the WT and *lpcat* across treatments. (e) Normalized expression counts of DEGs associated with the endomembrane trafficking system.

To seek insight into the molecular mechanism underlying the *lpcat* root phenotype, we conducted an RNAseq analysis of roots raised in the presence or absence of lysoPAF, and in combination with ONO. Pairwise comparisons between the *lpcat* mutant and the WT were conducted to identify differentially expressed genes (DEGs). A total of 153 DEGs were found in the roots between the *lpcat* mutant and the WT when grown on MS plates, of which 141 were up-regulated, and 12 were down-regulated. The number of DEGs rose to 666 when treated with lysoPAF, among which 408 were elevated and 258 were suppressed. The number of DEGs was reduced to 279 when grown on plates supplemented with lysoPAF plus ONO, of which 64 were down-regulated and 215 were up-regulated (Fig. 5a, Supplementary Data S2). Venn diagrams showed that 245 and 224 genes were up-regulated and down-regulated, respectively, under lysoPAF treatment (Fig. 5b and c). The DEGs could be categorized into six clusters (Fig. 5d). KEGG enrichment showed that a number of up-regulated genes are involved in protein processing in the ER, lipid metabolism, and phenylpropanoid biosynthesis (Fig. 5d).

The root transcriptome of the *lpcat* mutants features many genes associated with the unfolded protein response (UPR) and ER stress (Fig. 5e). The UPR is mediated predominantly through the transcription factor basic leucine zipper 60 (bZIP60) signaling pathway, in which bZIP60 regulates the expression of ER stress-responsive genes (Li et al. 2020). Notably, the bZIP60 transcript levels were elevated in the *lpcat* roots even in the absence of lysoPAF treatment. The addition of lysoPAF elevated the bZIP60 transcripts in the WT, but the degree of induction was much greater in the *lpcat* mutant. Several other regulatory targets of the bZIP60 pathway, including *ANAC103* (Sun et al. 2013), *ERDJ3A* (Song et al. 2015), *DCD*, and *CDC48D*, were also upregulated in the *lpcat* mutant compared with the WT, irrespective of with or without lysoPAF. These results indicate that the ER environment of the *lpcat* mutant was sufficiently attenuated to activate the IRE1/bZIP60 pathway and was further degenerated upon the supplementation of lysoPAF.

The root transcriptome of the *lpcat* mutant also unveiled perturbations in endomembrane trafficking and protein traffic at the ER/Golgi interface. These included the induction of Prenylated Rab Acceptor 1 (PRA1) domain protein genes, *AtPRA1.B5*(*AT5G01640*) and *AtPRA1.F1*(*AT1G17700*), which are receptors for Rab GTPase (Figueroa et al. 2001), as well as *SEC1B*(*AT4G12120*), which interacts with SNARE complexes and contributes to membrane fusion (Karnahl et al. 2018, Luo et al. 2022). AtARFB1α (*AT2G15310*), a member of the ARF GTPase family that functions in GTP-binding during vesicle coating, was significantly induced in the *lpcat* mutant when treated with lysoPAF. Similarly, AP180 (*AT1G05020*), anepsin-N-homolog (ANTH) protein involved in clathrin coat assembly and vesicle budding from membranes, was also induced in the *lpcat* mutant (Barth and Holstein 2004). Protein trafficking from the ER to the Golgi is regulated by the coat protomer complex II (COPII) (Marti et al. 2010). COPII vesicles are formed by a set of proteins, including Sar1, Sec23/24, and Sec13/31, which assemble on the surface of the ER at specialized regions known as the ER export sites (Chung et al. 2016). Our RNA-seq data show that both *SAR1a* (*AT1G09180*) and *SEC31A* (*AT1G18830*) were upregulated in the *lpcat* mutant (Fig. 5e).

## Discussion

The homeostasis of LPC is inseparable from that of PC remodeling, due primarily to the ubiquitous operation of the Lands cycle. In this study, we focused on the critical role of lipid remodeling in LPC homeostasis and uncovered phenotypes of the *lpcat* mutant that strongly suggest its involvement in membrane maintenance and root growth. That changes in the steady-state level of LPC are the main metabolic footprint in the *lpcat* mutant makes it a suitable system to dissect the functional significance of LPC, and the fine-tuning of the LPC/PC ratio in plant cells. However, exogenous LPC application yielded no apparent phenotypic differences between WT and the *lpcat* mutant, although a high concentration of 300 *μ*g/mL markedly inhibited root growth in the WT as well. Our assumption that factors relating to LPC breakdown buffered the effect of added LPC is supported by reports that multiple LysoPL isoforms, capable of deacylating LPC to release fatty acids and GPC, are present in Arabidopsis (Gao et al. 2010, Miao et al. 2019). Subsequent hydrolysis of GPC by glycerophosphodiester phosphodiesterase yields G-3-P and free choline (van der Rest et al. 2004). In support of this supposition, our previous study detected increased extracellular deposition of free choline in the water-soluble fraction of the *lpcat* mutant (Wang et al. 2012). Thus, in this study, we supplemented MS medium with lysoPAF, a structural analog of LPC that resists lysoPL deacylation, to create a chemical phenotyping condition in which lysophospholipid levels remain stably elevated in plant cells. Using this setup, we examined the impact of LPCAT loss on root growth. The *lpcat* mutant exhibited sensitivity to lysoPAF compared with the WT (Fig. 2a and b), and the introduction of functional LPCAT (LPCAT-EGFP) restored root growth and overcame the lysoPAF inhibition observed in the *lpcat* mutant (Fig. 4c) (Supplementary Fig. S3). Microscopic examinations revealed pronounced cellular morphological changes under lysoPAF treatment (Fig. 2d) that indicate an early and accelerated progression of vacuole maturation in the *lpcat* mutant during root cell differentiation. In plant cells, the vacuolar membrane is derived primarily from the ER, particularly during early differentiation (Viotti et al. 2013). The vacuolation process must be properly regulated during growth, as premature or excessive vacuole maturation can disrupt the balance between cell division and elongation, and impair overall root development. The most plausible interpretation of the root growth and cellular phenotypes is that LPCAT attenuates the cellular effects of lysoPAF by acylating it to PAF, a cylindrical lipid similar to PC.

When lysoPAF and ONO were applied together, ONO markedly suppressed the lysoPAF-induced phenotype in the *lpcat* mutant and restored normal root growth (Fig. 3). Although the exact mode of action of ONO, a phospholipase A₂ antagonist, remains unclear in plant cells, a likely explanation consistent with the data is that reduced PLA₂ activity in the *lpcat* background limits PC deacylation and, thereby, decreases the accumulation of lysophospholipids. This hypothesis warrants future validation through the quantification of LPC and PC levels under lysoPAF treatment.

We verified that both LPCAT1 and LPCAT2 are localized to the ER (Fig. 4). The ER is the central hub of membrane lipid synthesis, protein folding, and vesicular trafficking (Prinz 2010). Our results in the *lpcat* transcriptome changes suggest that disruptions in LPC acylation trigger broader cellular stress adaptation mechanisms in the ER, particularly the UPR, which is a conserved signaling pathway that maintains protein-folding homeostasis. In addition to the accumulation of unfolded or misfolded proteins, the UPR can also be triggered by alterations in the lipid composition of the ER membrane, a condition referred to as lipid bilayer stress. Membrane phospholipid saturation has been shown to be a factor in activating the UPR, and the Arabidopsis desaturase mutant fad2-1 displays hypersensitivity to chemically induced ER stress (Nguyen et al. 2019). In contrast, mutants of the non-specific phospholipase C3 exhibit reduced sensitivity to tunicamycin-induced ER stress, with evidence suggesting that phosphocholine production contributes to ER stress tolerance (Ngo et al. 2024). A decrease in PC levels and a perturbed PC/PE ratio in the membrane have been linked to ER stress and UPR activation in several organisms (Ho et al. 2020). The *lpcat* mutant does not display a significant change in the steady PC level in comparison with the WT, nor any apparent change in the PC/PE ratio. Our results extend previous findings on lipid composition and ER stress, and strongly suggest that the Lands cycle contributes to ER stress responses, and possibly to endomembrane trafficking, by regulating the LPC/PC ratio.

When grown under standard conditions, the *lpcat* mutant does not exhibit any apparent above-ground growth phenotype (Wang et al. 2012), contrasting with that of the lysophosphatidylethanolamine acyltransferase (LPEAT) mutant, which displayed defects in both vegetative growth and seed set (Jasieniecka-Gazarkiewicz et al. 2017). An important distinction between the *lpeat* and *lpcat* mutants is that the *lpeat* mutant had a drastic reduction in the relative and absolute amounts of phosphatidylethanolamine (PE), which is a major membrane lipid (Jasieniecka-Gazarkiewicz et al. 2017), while there was no reduction in the level of major phospholipids in the *lpcat* mutant. More importantly, LPEATs are structurally a different class of *sn-2* acyltransferases, closely related to lysophosphatidic acid acyltransferases of the Kennedy pathway for *de novo* glycerolipid synthesis (Stålberg et al. 2009). LPCATs, on the other hand, are members of the MBOAT family and are structurally more resembling the diacylglycerol acyltransferase 1 (DGAT1) (Zou et al. 1999). Consequently, there could be important distinctions in membrane localization between LPEAT and LPCAT. The *lpcat* mutant displayed an elevated LPE level as well, a metabolic outcome consistent with Arabidopsis LPCATs exhibiting broad lysophospholipid substrate specificity including for LPE (Stahl et al. 2008). However, the changes in lysophospholipids in the *lpcat* mutant were much less pronounced, whereas the *lpeat* mutant showed an astonishing 10- to 15-fold increase in LPC levels. These differential effects may reflect the distinct roles of the two classes of enzymes. LPEAT functions primarily in the *de novo* phospholipid biosynthetic pathway, while LPCAT participates mainly in lipid remodeling. Although the *de novo* pathway provides the structural foundation for membrane biogenesis, our results strongly suggest that the phospholipid remodeling pathway ensures the functional integrity and adaptive responsiveness of membranes. In addition to our previous finding that LPCAT participates in metabolic interactions between the *de novo* glycerolipid biosynthesis pathway and the Lands cycle (Wang et al. 2012), the present study reveals that LPCAT also functions as a modulator of ER membrane structure by converting LPC to PC, thereby maintaining lipid compositional balance and proper endomembrane trafficking. These findings highlight the importance of lipid homeostasis as a structural determinant of membrane organization and underscore the broader roles of lipids in regulating cellular responses.

## Materials and Methods

### Plant materials

The *Arabidopsis lpcat* mutant used in this study is the *lpcat1/lpcat2–2* mutant (Col-0 background) previously described (Wang et al. 2012). Transgenic lines expressing 35S::EGFP-LPCAT, 35S::SP-EGFP-LPCAT, 35S::LPCAT-EGFP-CT, and 35S::LPCAT-EGFP were produced in this study. The *Arabidopsis* lines tagged with fluorescent proteins used in this study included CD3–968 (Nelson et al. 2007) and HDEL-CFP.

### Plant growth and treatments

*Arabidopsis* plants were grown at 22–24 °C with a 16-h light/8-h dark photoperiod at 120–150 mE.m^−2^.s^−1^. In all germination experiments, the *Arabidopsis* seeds were cold stratified for 3 days at 4 °C to synchronize germination. Sterilized seeds were germinated on half-strength MS media containing various concentrations of lysoPAF (Avanti Polar Lipids), ONO-RS-082 (Endo life sciences), or LPC (Avanti Polar Lipids). The seeds were dispensed at an intensity of around 60 seeds per 25 mL of medium in square Petri dishes with grids (VWR International). The primary root length was measured using ImageJ (NIH, Bethesda, MD, USA).

### Microsomal preparation and LPCAT enzyme activity assay

The microsomal preparation and LPCAT enzyme activity assay were performed as described by Wang et al. (2012). In brief, about 1 g of root tissue from 5d-old *lpcat* mutant and WT seedlings was used to isolate microsomal fractions. The lysophospholipid acylation enzyme assay was conducted in 1.0 mL of 0.1 M potassium phosphate buffer, containing 6 *μ*M [14C]-oleoyl-CoA (20 nCi nmol^−1^) and 200 *μ*M 18:1-LPC. Reactions were initiated by adding 60 mg of microsomal protein to the pre-warmed mixture at 30 °C, and were stopped after a 4-min incubation, by adding a mixture of acetic acid/H_2_O (5:95 v/v) and chloroform/methanol (2:1 v/v). Lipid extracts were then separated by thin-layer chromatography (TLC) on silica gel 60 plates (EMD Millipore Corporation, Germany, http://www.emdmillipore.com), with a solvent system of chloroform/methanol/acetic acid/H_2_O (90:15:10:3 v/v/v/v). The PC species was scraped from the TLC plates, and its radioactivity was measured.

### Generation of green fluorescence fusion protein constructs

The DNA fragment encoding the enhanced green fluorescent protein, the *EGFP* gene, was amplified from the pK7WGF2 vector. An expression cassette (GCGGCCGCNNNNNNCCATGG/CCCGGGNNNNNNGGCGCGCC, with the *Not*I site at the 5′ end, the *Nco*I/*Xma*I sites in the middle, and the *Asc*I site at the 3′ end underlined) was designed to connect the *EGFP* and *LPCAT* genes. For the N-terminal EGFP-LPCAT fusions, the EGFP coding region without a stop codon was amplified with primers JW219, incorporating *Not*I site and JW220 incorporating *Nco*I site. Full length *LPCAT1* was amplified with primers JW221 incorporating *Nco*I site/JW217 incorporating *Asc*I site, and *LPCAT2* was amplified using primers JW222 incorporating *Nco*I site/JW218 incorporating *Asc*I site, respectively, to produce continuous open reading frames with *EGFP*. For the C-terminal LPCAT-EGFP fusions, the *LPCAT* cDNA fragments without stop codons were amplified with primers JW160 incorporating *Not*I site/JW161 incorporating *Xma*I site for *LPCAT1* and JW162 incorporating *Not*I site/JW164 incorporating *Nco*I site for *LPCAT2*, respectively. The *EGFP* fragment was then amplified with primers JW148 incorporating *Xma*I or *Nco*I site and JW149 incorporating an *Asc*I site to generate a continuous open reading frame with the *LPCAT*s. The LPCAT-EGFP-CT fusions were constructed by overlapping PCR with designed primers using LPCAT-EGFP as a template. The resulting cassettes were subcloned between the *Not*I and *Asc*I sites of the entry vector pER367 (Wang et al. 2012), respectively. The obtained pER367 vectors, harboring the above expression cassettes, were recombined with the destination vector pER330 under the control of the cauliflower mosaic virus 35S promoter to generate different types of EGFP fusion plasmids. All of the primers used above are shown in Supplementary Table S1.

### Arabidopsis transformation

For stable transformation, plants were transformed by the floral dip method (Clough and Bent 1998) using *Agrobacterium tumefaciens* GV3101.

### Yeast complementation assay

Six fragments containing EGFP-LPCAT, LPCAT-EGFP, and LPCAT-EGFP-CT were amplified from the above EGFP fusion constructs and inserted into the pYES2.1 vector (Invitrogen), respectively. Each plasmid was introduced into the yeast *lpcat* mutant *∆lca1* as previously described (Chen et al. 2007). Yeast cells were grown in 15 mL of SC-Ura medium containing 2% glucose overnight and were then transferred to the expression induction medium SC-Ura + 2% galactose and 1% raffinose for 12 h. Cultures were diluted to OD_600_ values of 0.5, 1, and 2. For each dilution, 5 *μ*L of the culture was spotted on SC-Ura plates containing 20 *μ*g/mL of lysoPAF and then incubated at 28 °C for 24–36 h.

### Light microscopy

Root tissues were collected from 5-day old seedlings growing vertically on half-strength MS medium containing various concentrations of lysoPAF. After rinsing with a phosphate buffer (pH 6.8) briefly, the roots were cut into 0.5-cm long and fixed with 2.5% glutaraldehyde overnight at 4 °C, and they were then postfixed in 1% osmium tetroxide (Electron Microscopy Sciences) for 8 h at 4 °C. The samples were dehydrated in a graded ethanol series and embedded in Epon 812 resin (Electron Microscopy Sciences). For light microscopy, semi-thin sections were sliced into 1–2 *μ*m and stained with a 1% toluidine blue O solution.

### Confocal microscopy

For the colocalization of CFP and GFP, excitation lines of 405 nm for CFP and 488 nm for GFP were used with the multi-track of the microscope. Bandpass filters of 475/525 nm and 505/530 nm were used for detecting CFP and GFP, respectively. For the colocalization of GFP and mCherry, excitation lines of 488 nm for GFP and 543 nm for mCherry were used alternately with the multi-track of the microscope. Bandpass filters of 505–530 nm and 560–615 nm were used to detect GFP and mCherry, respectively. Images were analyzed with the image processing software LSM Image Browser or ImageJ.

### Analysis of fatty acid composition and lipidomic profiles

Total fatty acids were extracted from ~30 mg of fresh roots, and the fatty acid composition was determined by gas chromatography with heptadecanoic acid (17:0) as a quantitative internal standard. Lipidomics analysis was performed according to the protocol from the Kansas Lipidomics Research Center (http://www.k-state.edu/lipid/lipidomics; Welti et al. 2002). Briefly, root tissues collected from 5-day-old seedlings were heated in 2 mL of isopropanol with 0.01% butylated hydroxytoluene at 75 °C for 10 min to inactivate the lipases. Chloroform and methanol (2:1, v/v) were added for extraction. After several extractions, the combined extracts were washed with 1 M potassium chloride to remove proteins and carbohydrates. The chloroform phase was taken out and dried under a nitrogen stream. The lipid extracts were dissolved in chloroform for lipidomic analysis. Electrospray ionization tandem mass spectrometry (ESI-MS/MS) analysis was performed with five biological replicates for each growth condition at the Kansas Lipidomic Research Center. Statistical significance was determined using a two-tailed Student’s *t*-test.

### RNA sequencing and differential expression analysis

Total RNA from the roots of 5-day-old seedlings on 1/2 MS media (control), or 1/2 MS media containing 20 *μ*g/mL of lyso-PAF were extracted using a Plant RNA Isolation Mini Kit (Agilent, https://www.agilent.com/). The yield and RNA purity were determined spectrophotometrically with a Nanodrop 1100 (Thermo Fisher Scientific), and the quality of the RNA was verified by an Agilent 2100 Bioanalyzer (Agilent). Purified total RNA was precipitated and resuspended in RNase free water to a final concentration of 100 ng/*μ*L. Eighteen cDNA libraries were constructed using the TruSeq RNA Sample Preparation Kit v2 (Illumina) with three replicates for the WT and the *lpcat* mutant at each growth condition, respectively. Paired-end sequencing was conducted on the Illumina HiSeq2500 (Illumina), generating 101-nucleotide reads, at the National Research Council Canada, ACRD-Saskatoon, Canada. Raw data processing was performed as described previously (Li et al. 2017). Differential expression analysis was performed with DEseq2 between the mutant and wild-type at each condition (Love et al. 2014) (Supplementary Data S1). Normalized counts from DESeq2 were expressed as gene expression levels. Genes with less than five reads across all samples were excluded to eliminate the extremely low-expressed transcripts. Hierarchical clustering was performed with normalized counts using the *hclust* in R. GO enrichment analysis and KEGG enrichment were performed with *clusterProfiler* in the R program (Yu et al. 2012) (Supplementary Data S2). Genes involved in secretory pathways were categorized (Bassham et al. 2008, Heard et al. 2015) (Supplementary Data S3). Lipid metabolism genes were categorized according to the list from the Arabidopsis Acyl-Lipid Metabolism database (http://aralip.plantbiology.msu.edu) (Supplementary Data S4). Functional annotations of genes were obtained from the Bio-Array Resource for *Arabidopsis* Functional Genomics database (BAR) (http://bar.utoronto.ca/) using the AGI numbers.

## Supplementary Material

Supplementary_Figs_S1-S3_pcaf174

Supplementary_Table_S1_pcaf174

Supplementary_Data_S1_pcaf174

Supplementary_Data_S2_pcaf174

Supplementary_Data_S3_pcaf174

Supplementary_Data_S4_pcaf174

## Data Availability

The data underlying this article are available in Gene Expression Omnibus under the accession GSE128799.
