# Supplementary material for: Lysophosphatidylcholine homeostasis via the Lands cycle regulates root growth in Arabidopsis
Source: Plant Cell Physiol. 2026 Jan 12;67(5):467–78. doi: 10.1093/pcp/pcaf174 (PMC13227155; doi:10.1093/pcp/pcaf174)
Supplement: Supplementary_Figs_S1-S3_pcaf174 [file supplementary_figs_s1-s3_pcaf174.pdf]

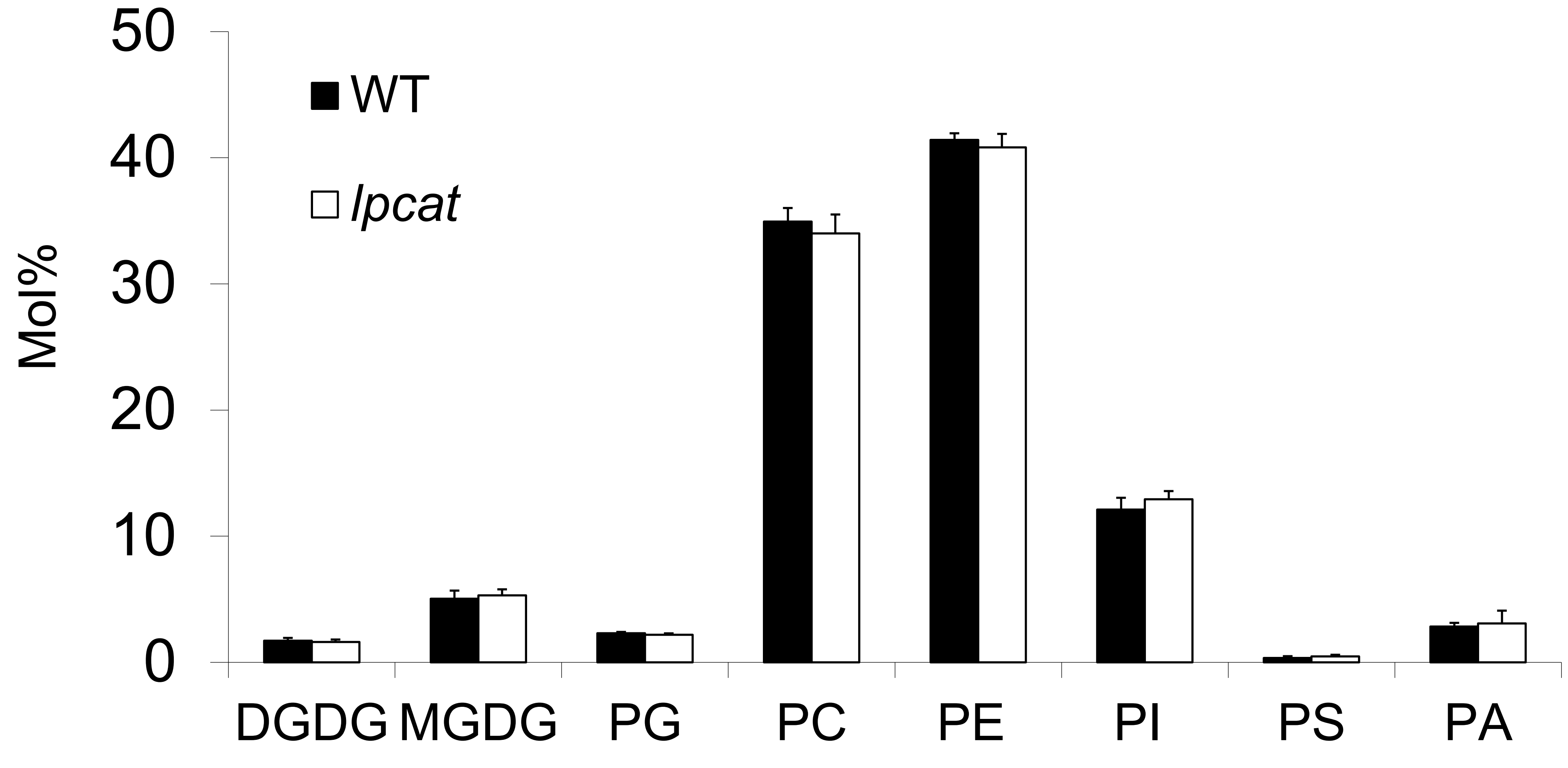

Supplementary Fig. S1. Glycerolipid composition in *lpcat* roots.

Relative percentages of major glycerolipids in *lpcat* and WT roots. Lipid classes: DGDG, digalactosyldiacylglycerol; MGDG, monogalactosyldiacylglycerol; PG, phosphatidylglycerol; PE, phosphatidylethanolamine; PI, phosphatidylinositol; PS, phosphatidylserine; PA, phosphatidic acid. Data represent means ± SD of five independent biological replicates. Asterisks indicate statistically significant differences relative to WT ($P < 0.05$; Student's *t*-test).

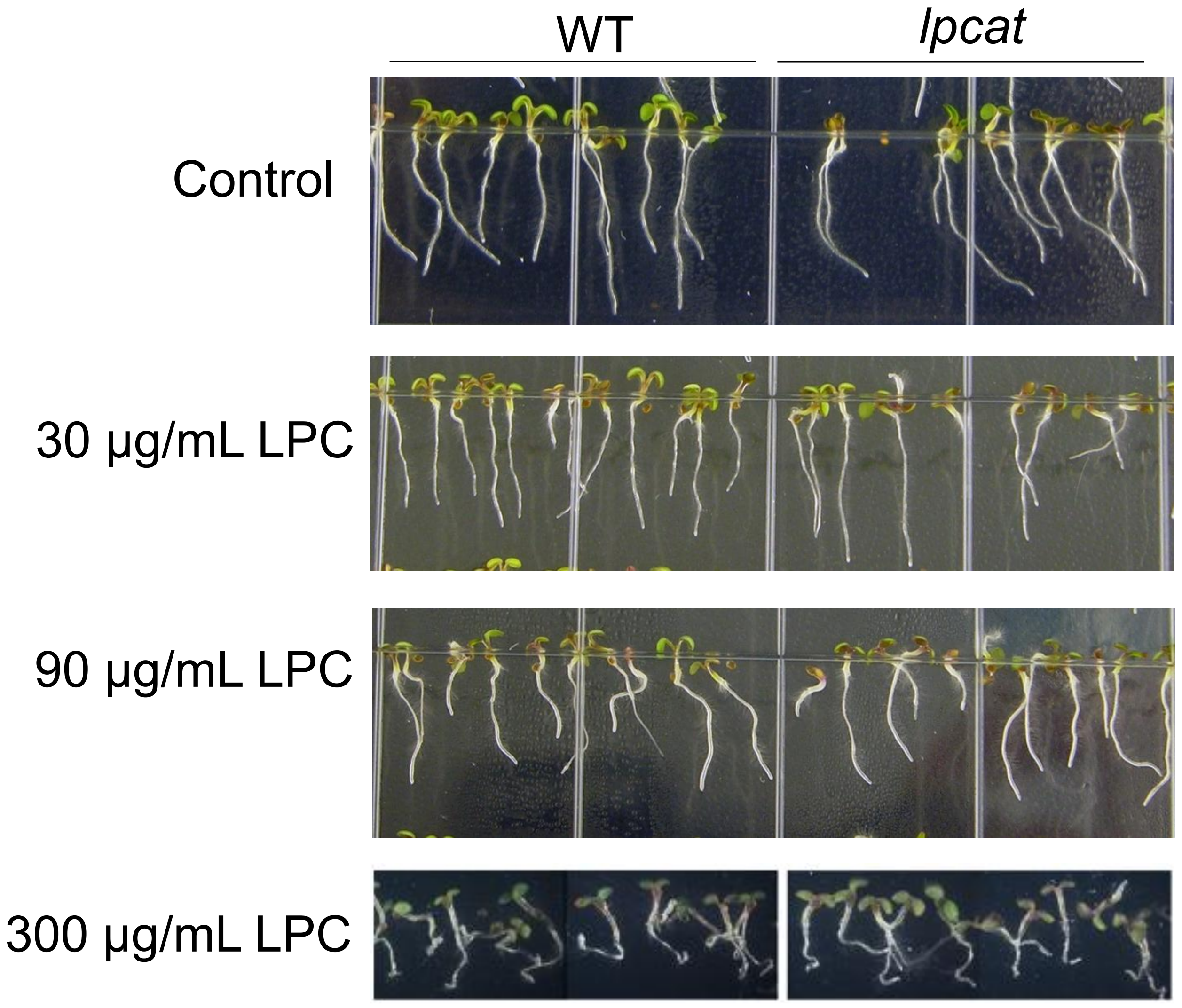

Supplementary Fig. S2. Effect of exogenous LPC supplementation on *lpcat* mutant root growth.

Seedlings were grown on media supplemented with 30, 90, or 300 μg/mL LPC, and root growth was assessed after 5 days of germination. Representative images are shown.

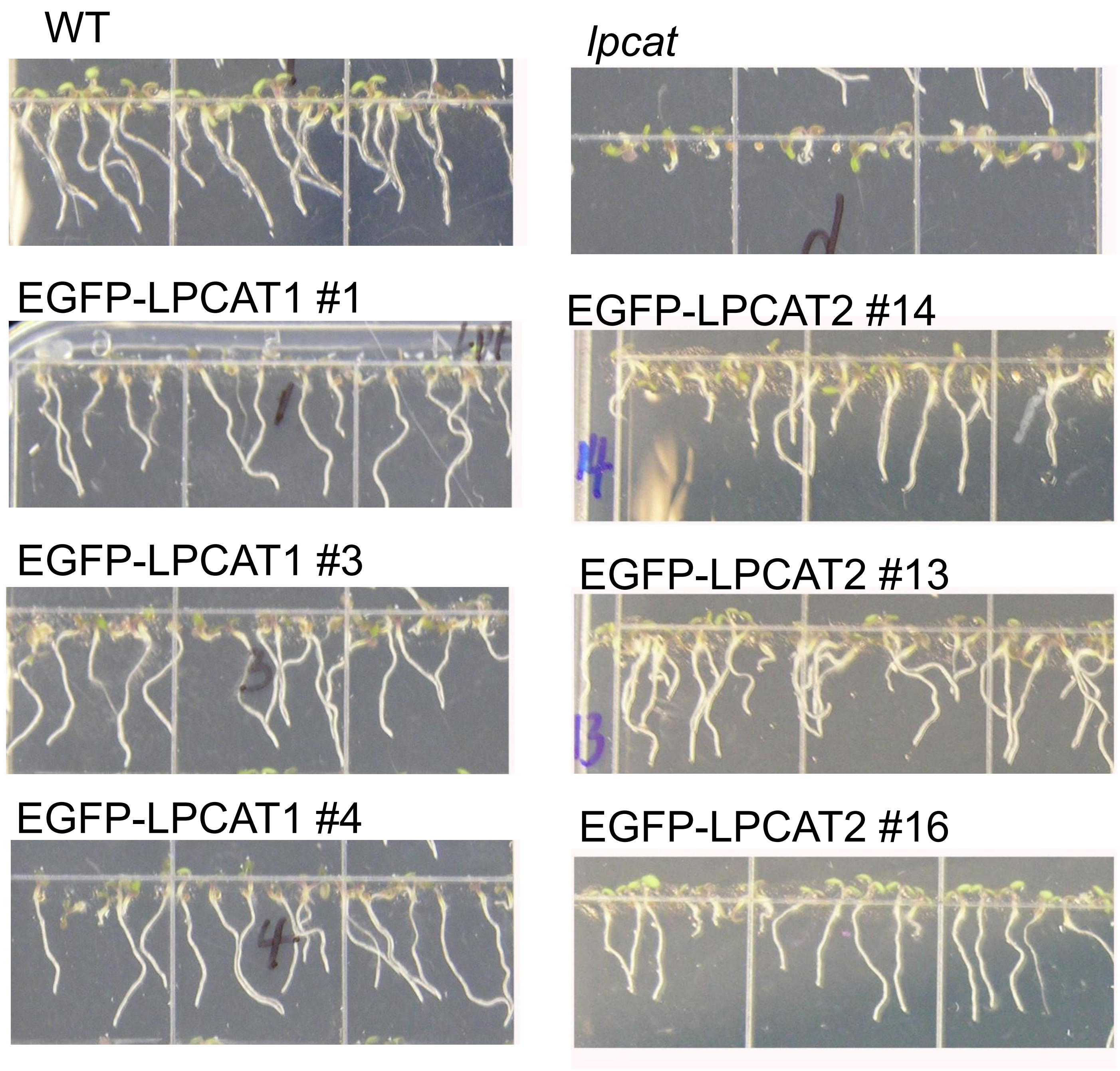

Supplementary Fig. S3. Effect of EGFP-LPCAT1 or EGFP-LPCAT2 expression in the *lpcat* mutant on lysoPAF sensitivity.

Root growth of *lpcat* seedlings expressing EGFP-LPCAT1 or EGFP-LPCAT2 was evaluated in the presence of 35 µg/mL lysoPAF. For each construct, three independent homozygous lines were analyzed.
